# Implementing a Cancer Fast-track Programme between primary and specialised care in Catalonia (Spain): a mixed methods study

**DOI:** 10.1038/bjc.2011.308

**Published:** 2011-08-09

**Authors:** J Prades, J A Espinàs, R Font, J M Argimon, J M Borràs

**Affiliations:** 1 Catalonian Cancer Plan, Duran i Reynals Hospital, Av. Gran Via de l’Hospitalet 199–203, Hospitalet de Llobregat, Barcelona 08908, Spain; 2 Health Service Procurement & Assessment, Catalonian Health Service (Servei Català de la Salut), Travessera de les Corts 131–159, Barcelona 08028, Spain; 3 Clinical Sciences Department, Bellvitge Biomedical Research Institute (Institut d’Investigació Biomèdica de Bellvitge – IDIBELL), University of Barcelona, Av. Gran Via de l’Hospitalet 199–203, Hospitalet de Llobregat, Barcelona 08908, Spain

**Keywords:** programme effectiveness, quantitative evaluation, qualitative research

## Abstract

**Background:**

The Cancer Fast-track Programme's aim was to reduce the time that elapsed between well-founded suspicion of breast, colorectal and lung cancer and the start of initial treatment in Catalonia (Spain). We sought to analyse its implementation and overall effectiveness.

**Methods:**

A quantitative analysis of the programme was performed using data generated by the hospitals on the basis of seven fast-track monitoring indicators for the period 2006–2009. In addition, we conducted a qualitative study, based on 83 semistructured interviews with primary and specialised health professionals and health administrators, to obtain their perception of the programme's implementation.

**Results:**

About half of all new patients with breast, lung or colorectal cancer were diagnosed via the fast track, though the cancer detection rate declined across the period. Mean time from detection of suspected cancer in primary care to start of initial treatment was 32 days for breast, 30 for colorectal and 37 for lung cancer (2009). Professionals associated with the implementation of the programme showed that general practitioners faced with suspicion of cancer had changed their conduct with the aim of preventing lags. Furthermore, hospitals were found to have pursued three specific implementation strategies (top-down, consensus-based and participatory), which made for the cohesion and sustainability of the circuits.

**Conclusion:**

The programme has contributed to speeding up diagnostic assessment and treatment of patients with suspicion of cancer, and to clarifying the patient pathway between primary and specialised care.

Concern about the delay in diagnosis of cancer and its clinical and psychosocial implications for patients has led a number of European health systems to develop fast-track referral mechanisms for patients with suspicion of cancer. In Catalonia, the Cancer Fast-track Programme (CFP) was launched in 2005 for neoplasms registering the highest incidence and mortality rates. The incidence of breast, colorectal and lung cancer accounts for 43% of all cancer incidence in women (28.1%,15.2% and 2.4%, respectively) and 30% in men (13.9 and 16.1% in lung and colorectal cancer) in Catalonia (Borràs *et al*, 2008). The CFP's aim was therefore to reduce the time that elapsed between well-founded suspicion of cancer and the start of initial treatment. To this end, a time point (30 days) was set as a policy target for all patients included in this special pathway, bearing in mind that there was no universally predefined standard, and provided in all cases that the characteristics of the specific diagnosis process allowed for this.

Despite the controversy surrounding such a programme's capacity to affect survival (Myrdal *et al*, 2004; Ramos *et al*, 2007), as initially suggested by the English National Health Service (NHS Executive, 2000), aims other than the positive perception that patients, professionals and policymakers have of a programme of this type were also involved. On the one hand, better health-care quality was sought, by reducing the variability in suspected-cancer referral in primary care and achieving more standardised health-care processes in secondary care; and on the other, the reduction in waiting time was assumed to entail a reduction in the possible psychosocial impact on the patient caused by a period of intense anxiety and a sensation of vulnerability intervening between suspicion of cancer, definitive diagnosis and the start of treatment, a relationship supported by a number of studies (Risberg *et al*, 1996; Hislop *et al*, 2002; Barton *et al*, 2004; Cornford *et al*, 2004; Brett *et al*, 2005). A relevant difference vis-à-vis other European experiences is that the development of the CFP includes the treatment component, with the aim of preventing the imbalance between greater diagnostic speed and problems that arise after access to therapeutic resources, such as operating-theatre ‘bottle-necks’, a frequent situation for fast-track procedures limited to diagnosis (Chohan *et al*, 2005; Rai and Kelly, 2006; Olesen *et al*, 2009).

The Catalonian Health Service (CHS) is a universal health-care system organised on the basis of the separation between public funding/sponsorship and a mix of private- and publicly-owned health facilities (foundations, consortia, and church- or publicly-run centres). It was this context that led the Department of Health to permit circuits to be freely organised, subject in all cases to the use of the referral guidelines agreed by experts from both care levels, and the setting-up of a system of monitoring indicators.

This study analysed the overall effectiveness of the system, using data sets that focused on the implementation of the colorectal, breast and lung cancer fast tracks in Catalonia. It was considered necessary to combine a quantitative assessment of programme-monitoring data with a qualitative assessment of health professionals’ and administrators’ perception of the organisational change entailed by the programme's implementation. The policy knowledge yielded by this analysis is expected to improve the second phase of implementation, during which bladder and prostate cancers are scheduled to be added to the programme. Quantitative and qualitative methods stress the holistic and wide-ranging approach needed to understand an innovative programme, thanks to which health-service delivery has changed.

## Materials and methods

**Figure 1:**
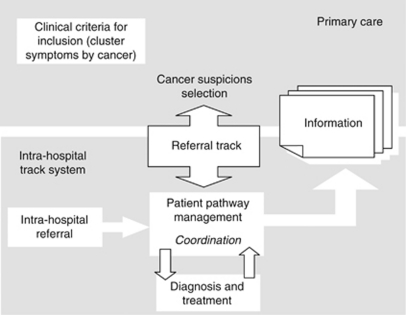
Scope and functions of the CFP.

The CFP was proposed as a means of reducing the lag (time elapsed) between well-founded suspicion, diagnosis and treatment of cancer, by designing circuits that would foster the rapid coordination of the process circuit (see Figure 1). Similarly, the health-care authorities had to issue some organisational recommendations for effective implementation of these circuits, for example, clinician responsible for disease, definition of maximum waiting times for diagnosis, study without hospitalisation where possible or coordination mechanisms in the event of referral to another hospital. Furthermore, the development of the programme implied the need to set up an information system, so that it could be assessed on the basis of common indicators for the entire CHS. Each hospital was required to submit aggregate data on patients that entered into its circuit, with the recording and analysis of this information being covered by programme funding, subject to incentives in the prospective annual contract between the CHS and the individual hospital. Three possible origins of suspicions for routing fast-track patients were laid down, namely, primary care, emergencies, or other clinical departments in routine monitoring or screening. Detection meant that the patient in question had to be referred to a ‘rapid diagnosis unit’ at a teaching hospital present in each health region (decentralised health authorities of the CHS). For study purposes, a fast-track circuit was defined as a process that envisages a systematic approach to a complex pathology involving two or more levels of care and several clinical departments. A CFP seeks to synchronise the clinical needs of a potential cancer patient, by implementing passive (e.g., slots in diagnostic tests) or active measures (e.g., case management throughout the care process), ultimately leading to improved coordination.

### Quantitative assessment

With regard to quantitative data sources, the following seven fast-track monitoring indicators were laid down: number of patients included in the CFP; cancer patients diagnosed through the CFP route; patients referred from general practitioners (GPs); compliance with referral guidelines; cancer detection rate; mean time between detection of suspected cancer and start of treatment; and distribution of the wait among different categories (‘over 30 days’, ‘30 to 45 days’ and ‘over 45 days’). Our analysis covered the period 2006–2009. The hospitals were tasked with recording and sharing these data with the primary-care level. Hospitals have reported quantitative data to the CHS on an annual basis since 2006, with the health regions having the obligation of ensuring the validity of the data. Some clarifications must be made with regard to data registration criteria. All new cancer diagnoses were included but cases of relapse were excluded. Date of entry into the circuit coincided with the referral date, and date of start of ‘treatment’ with the first treatment applied to the patient instead.

### Qualitative evaluation

Qualitative study consisted of semistructured interviews conducted from January to March 2009, with health professionals drawn from primary and specialised care, and planning staff from the health regions.

### Data sources

A total of 83 health professionals were recruited from 18 fast tracks. Professionals were deemed eligible if they participated in fast-track management as stakeholders or data managers. For selection of informants and composition of the purposive sample, three inclusion criteria were established. To ensure that the views of different health regions could be explored, the first criterion ensured the presence of at least one fast track (primary and specialised care) per health region. The second criterion reinforced a systematic approach to the phenomenon under study, by insisting on the presence of informants from each fast track reflecting professional views, whether institutional, technical or process-related (see Table 1). A final criterion to be considered was the participation of different levels of hospitals, something that is both appropriate and pertinent bearing in mind the usual fast-track split between district and tertiary hospitals (e.g., thoracic surgery in lung cancer). Participants were interviewed by an experienced, qualitative researcher. The implementation of the study began through contact with the hospital and local primary-care directors, who proposed a short list of health professionals selected in accordance with our criteria. The resulting list was endorsed by the health regions. The health professionals concerned were then sent a letter of invitation explaining the research goals, together with a confidentiality agreement. The consent form was formally signed at the meeting. No one refused the invitation to participate.

### Data-collection procedures

A semistructured interview ensured that all critical points were addressed. To elicit beliefs and experiences, participants were given the necessary flexibility to enable them to volunteer information on topics of relevance to them. The health professionals selected were interviewed on a one-to-one basis for 45–75 min at the hospital or primary-care offices. Interviews started with a general question on fast-track development and ended with a question on how to introduce future changes to improve the programme. No notes were taken by the researcher during the interview; instead, all interviews were audiotaped and transcribed in full. These data were then compiled into a documentary record and rendered anonymous to protect confidentiality. Every transcription was checked against its corresponding sound recording, and accuracy was found to be good. A preliminary analysis was conducted after each interview. All the professionals selected for study purposes consented to the interviews being recorded.

### Analysis

Interview data were examined inductively. This process allowed for data references to be labelled and developed for subsequent analysis. Grounded theory methodology was considered appropriate for describing fast-track implementation and organisation according to the professionals’ points of view. As our study was theoretical and aimed at incorporating the organisational context in which fast tracks had been implemented, we used axial coding, as described by Strauss and Corbin (1998). Data were electronically coded using the ATLAS.ti software programme (Muhr, 1997). Grounded theory enabled language use to be understood and professionals’ beliefs to be communicated; the constant comparison method ensured that recurring views and experiences were obtained. Thanks to the large number of interviews (83), ‘saturation’ of information was achieved (Guest *et al*, 2006). The need to manage this amount of data and look for patterns entailed to chart the coding data. While the coding process and emerging themes were derived from issues raised by participants, the properties and dimensions used to examine the data were drawn from comparative fast tracks in the scientific literature. Examples of codes were ‘dissemination of inclusion criteria in primary care’, ‘changes in the management of specialists’ diagnostic schedule’ and ‘case manager functions’. Coding/interpretation consistency was checked during analysis, by reviewing the transcripts at different points in time. To ensure the relevance and appropriateness of the categories (first interpretative stage), health professionals from different disciplines were then asked to give their considered opinion. Examples of categories used were ‘referring patients performance’, ‘weaknesses in feedback between levels of care’ and ‘hospital strategy for implementing the programme’. An explanatory framework was thus identified by focusing our analysis on patterns of the way in which action/interaction takes place in every circuit and is aligned with organisational conditions (e.g., setting a common gateway for suspected case referrals at hospitals). A specific effort was made to capture this stage of interpretation, that is, by mapping, creating typologies (Bryman and Burgess, 1993) and finding associations among themes.

## Results

### Quantitative monitoring

A total of 56 020 persons were included in the CFP during the period 2006–2009. The results of the monitoring indicators regarding circuit performance are presented in Table 2. The degree to which the programme had been implemented increased during the study period at the 65 hospitals in Catalonia, that is, while there were complete data on 74% of hospitals attending to cancer patients in 2006, this figure had risen to around 96% by 2009. The number of patients included in the programme increased for all three neoplasms. Half or more of all new patients with any of the three neoplasms were diagnosed via this pathway across the period.

The proportion of patients referred by GPs across the entire study period was ∼60% for colorectal versus 40–50% for breast and lung cancer. There is less variability among neoplasms in the case of adherence to the clinical criteria for inclusion of possible patients in the circuits, with the proportions being >70% in all three cases. The cancer detection rate showed a statistically significant moderate decreasing trend (*P*<0.001).

The results for the circuits in terms of waiting times are also shown. Mean time from detection of suspected cancer in primary care to start of initial treatment was under or a little over 30 days. Lung cancer displayed a variable trend, albeit somewhat distant from the 30-day target, something that is, in part, attributable to the complexity of the treatment process, inasmuch as this includes thoracic surgery concentrated at tertiary hospitals.

**Figure 2:**
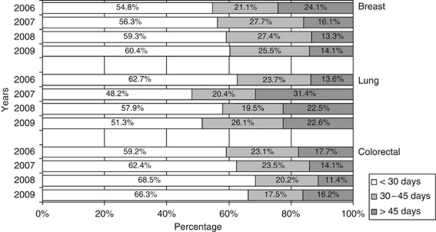
Waiting times in breast, lung and colorectal cancer (2006–2009).

Lastly, the distribution of cases was considered by reference to their proportions in terms of waiting times, specifically ‘over 30 days’ ‘30 to 45 days’ and ‘over 45 days’ (Figure 2). Breast cancer was the only type that displayed a clearly positive trend. Lung cancer registered variable figures, albeit worse overall; the data revealed that ∼50% of cases were divided between the two longest wait categories, with this split being largely proportional.

### Qualitative study

The CFP constitutes a formula for the virtual integration of different levels of health-care providers. Compared to the preceding situation, the implementation of circuits has meant a new orientation of cancer processes on the basis of two basic elements: on the one hand, primary care has a specific role vis-à-vis cancer by serving to detect suspected cases; and on the other, the organisation of the intrahospital track implies a loss of control of patient sources by clinical departments in favour of standardisation of flows and the diagnostic process.

#### Primary care

The creation of a specific track means that the way in which it is used acquires great importance. Initially, hospital professionals feared an overuse of the circuit in terms of referral of suspected cases by GPs, whether owing to inaccurate matches to guideline symptoms or inappropriate use of the route. The majority opinion, however, is that this has not taken place, which must, in good measure, be attributed to the set of actions undertaken to differing degrees by the various health centres (see Table 3). Indeed, of the circuits assessed, only one hospital was observed to be overwhelmed as a result of overindication (colorectal cancer).

The key to good acceptance of the programme in primary care lies, according to the physicians in this area, in having an instrument available that eliminates uncertainty (for both them and their patients) surrounding the length of the course of care for a potential cancer process. Indeed, the existence of these circuits has altered and standardised such physicians’ conduct in the face of suspicion of cancer. A total of 3 out of 12 GPs interviewed stated that, before the programme's introduction, it was a routine procedure to request a mammogram in any case where there was suspicion of breast cancer. Now, however, such a case would be referred immediately so as to prevent the processes being delayed any further by the health-care services.

In contrast, the feedback of clinical information from fast-tracked patients at the primary-care level, during or on conclusion of the diagnostic–therapeutic process, was viewed as deficient. Feedback showed itself to be extremely unsystematic and subject to the individual work practices of each hospital department. Indeed, it was very usual for GPs to call the hospitals to enquire about their patients’ progress. Furthermore, only 25% of the 18 fast tracks assessed had a software programme in place that allowed for the recording and multilateral consultation of clinical information based on suspicion of cancer, which would normally make for the smooth running of the interface at an operational level.

#### Specialised care

The intrahospital stage of the circuit encompasses notification of suspected-cancer cases, organisation of the patient pathway until final diagnosis, first treatment and feedback to primary care. Among the changes implemented (Table 3), mention should be made of identification of the ‘case management’ issue with the development of the programme, which took place at 13 of the 18 centres. Although case management was initially performed on seven occasions by the nurse, on three occasions by internal medicine and on three occasions by hospital administrative person monitoring, the nurse case–manager profile was acknowledged as being a success and indeed has been extensively implemented by most of the health centres in the period 2006–2009. As a consequence of such assessment, nurse case–managers exercised a key role in circuit cohesion, usually acting as the gatekeeper of suspected case referrals and the reference point for the patient and multidisciplinary team throughout the health-care process. The necessary degree of professional autonomy in the performance of their functions and poor delimitation of their scope of competence in some cases led to adjustment problems at some health centres.

The complexity inherent in the development of an ‘intrahospital circuit’ made the choice of programme implementation strategy extremely important. The logical premise expressed by the leaders was that the more the various stakeholders and policymakers were involved in co-designing and implementing the circuits, the greater the amount of objective changes that could be made to the health-care process and the greater the satisfaction that would be felt by health professionals regarding the previous situation. Three strategies were identified in the 18 circuits analysed:

- Top-down (*n*=5), where hospital management designed a flow chart and implementation programme in the territory, which was then transferred to the clinical teams.
- Consensus-based (*n*=10), where the clinicians responsible led the process of change, with the institutional support of hospital management.
- Participatory (*n*=3), where a leader was appointed and an implementation committee was set up, on which all the clinical departments were represented. The committee then proceeded to act on a horizontal, cross-department basis.

There are two elements of note in the assessment of the above strategies. On the one hand, the top-down and, in good measure, consensus-based strategies tended to limit the spread of knowledge of guidelines and intra-referral mechanisms among the clinical departments potentially involved, something that could negatively affect identification and effective referral of patients with suspicion of cancer. On the other hand, only the participatory strategy ensured an integrated development of the three diseases based on the rationale of a single circuit. Most of the interviewees were of the opinion that the logic of ‘three pathways, one circuit’ was prejudiced, when clinical and professional leaders who had to work together within the circuits were perceived as acting without a common reference framework, which occurred at 7 of the 18 hospitals analysed. Properly speaking, one is referring here to three ‘circuits’, that is, three distinct pathways, depending on the disease in question. Where there is such variability, organisational changes remain subject to the enthusiasm of the health professionals in charge of each disease. Indeed, some ‘circuits’ displayed an organisation that was substantially better than others, a situation that may make for inequities of care, in terms of the programme's overall effectiveness (impact on waiting times, cancer detection rate, etc.).

## Discussion

This assessment seeks to give an account of the implementation process of the fast-track cancer diagnosis and treatment programme, which was initiated in 2006 and has been positively assessed by health-care system stakeholders and policymakers as a whole. Half of all colorectal, lung and breast cancer patients are diagnosed today within periods of ∼30 days (longer in lung cancer) via a fast-track route created among care levels, which has made a considerable contribution to clarifying and accelerating cancer diagnosis and treatment. There will always be patients who cannot be diagnosed within the CFP but, on being incorporated once diagnosis has been made, they will nevertheless be able to take advantage of the enhanced speed to treatment and the greater degree of integration achieved by the entire patient pathway, with an implicit goal being to prevent the creation of a two-tier system in which routine referrals might be adversely affected in terms of waiting time (Jones *et al*, 2001). Evidence indicates that failure to recognise cancer symptoms in referral can double the wait to initiation of treatment (Pullyback *et al*, 2003). As Gomez *et al* (2010) conclude, ‘the kind of health-care system and the referral scheme (simple, clear, fast) for those suspected of cancer are relevant’.

One of the key aspects of the programme is regular assessment. Studies examining delays are difficult to compare because of the different end points used, for example, to start with, most are confined to the diagnostic component. Looking at the reference model for fast-track (cancer suspected) referral, namely, the English National Health Service's Two Week Rule (TWR) (NHS Executive, 1999), differences will be observed between this and Catalonia's experience in certain aspects. In the case of colorectal cancer, Thorne *et al* (2006) review grouped eight studies (period 2000–2003). Of the 2440 fast-tracked patients, 24.1% were diagnosed through the GP route and the cancer detection rate was 10.3%. Rai and Kelly (2006) grouped seven health centres (period 2003–2005) for a total of 1814 fast-tracked patients: in no case did the contribution of the TWR system to total colorectal cancer detection exceed 50%, the cancer detection rate ranged from 4.6 to 11%, and guideline adherence was 41–96%. Our study registered data which were significantly higher in terms of GP referral (around 60%) and cancer detection rates (around 30%), and similar in terms of adherence to referral guidelines. In breast cancer, the 5-year study (period 1999–2005) conducted by Singhal *et al* (2008) covered 6678 patients, 46.7% of the total seen in the period. The cancer detection rate was 16.4%, and guideline adherence was 71.9%. Our data were similar in terms of GP referral (42.8% in 2009) and guideline adherence, though the cancer detection rate was significantly higher (over 40% in all cases). One of the factors that might partly account for the difference in the detection rate is that the CFP not only encompasses cases originating in GP referral, but also those stemming from screening or emergencies. Also, the statistically significant decrease of the detection rate reflected the process of the program's implementation; to better assess this trend a longer period of time would be required. Experts are of the opinion that, while efforts in reducing the delay in lung cancer care do not impact on patient prognoses (Myrdal *et al*, 2004; Salomaa *et al*, 2005; González-Barcala *et al*, 2010), there is nevertheless convincing evidence to show that for some potentially curable patients delays at this point can decrease their chances of survival (O’Rourke and Edwards, 2000). Accordingly, it is essential that such efforts be sustained (Moody *et al*, 2004). Although there is no convincing evidence that rapid diagnosis of colorectal cancer improves prognosis (Ramos *et al*, 2007), a higher risk of death has nonetheless been observed when the delay is longer than 5 weeks in patients with suggestive symptoms (Tørring *et al*, 2011).

This study has some limitations. Insofar as the quality of the quantitative data is concerned, the statistical analyses were performed using the overall data available. Progressive implementation did not allow for an analysis of the data with a breakdown by hospital (e.g., in the case of the hospitals chosen for the qualitative study). A further limitation was the lack of a procedure for validating the data generated by the hospitals. Similarly, there is no regulation requiring hospitals to generate data on patients not included in the circuits, information that would enable one to ascertain whether such patients undergo a far longer diagnostic or therapeutic delay or are diagnosed at a more advanced stage of the disease. This also hinders comparison between the CFP and the performance of other fast-track processes. A clear limitation of the qualitative study lay in the selection process, which was based on proposals put forward by hospital managers and programme coordinators, and could have biased the choice of professionals towards those sensitive to the programme's effective implementation. The selection criteria specifically designed to target different profiles were intended to minimise this limitation. Our research focused on the views of professionals from 18 fast-track routes, thereby implicitly ruling out the possibility of capturing all the experiences and best practices that might exist in the health system as a whole.

Several important lessons were learned from this experience. The distinctive feature recognisable in the best hospital implementation strategies is their horizontal, cross-department mode of operation. From these hospitals’ experience, we know that it is essential not only to ensure a vertical approach to the process (accelerating it with new ways of channelling suspected cases between units, or relying upon on nurse case managers), but also to make provision for horizontal integration, especially among treatment hospital departments. At all events, caution must be exercised in establishing a causal relationship between implementation strategy in hospitals and quantitative results, as health-care organisation culture – which is hierarchical to a greater or lesser degree and guides individual behaviour within such organisations – tends to intervene in this relationship. Lastly, the implementation of the CFP involves an exercise in prioritisation for health administrators, that is, on the one hand, the inclusion of the first treatment within the 30-day reference period places a certain degree of stress on the management of operating-theatre schedules and the availability of certain health professionals, something that is also borne out by a qualitative study (Cornford *et al*, 2004); and on the other, the programme incentive system envisaged by the health authorities can sometimes clash with those already introduced for other illnesses. The result is a certain degree of ‘competition’ among diseases at general hospitals.

Prompt diagnosis and treatment of patients with suspicion of cancer has, in essence, amounted to a commitment to the generalisation of process-based management among care levels. This change was in contrast to the initial scepticism of health professionals, who saw it as simply formalising something that had already existed informally. However, this has led to a marked qualitative leap in their opinion, though no data are available on delay times before programme implementation. Despite the implementation problems outlined here, the circuits have succeeded in impacting positively on health service delivery, something that needs to be weighed on the basis of improving monitoring indicators and providing data on patients not included in the circuits. This would give us a valuable knowledge in terms of potential delays in other cancer patients. At all events, unnecessary or unexplained delays are now being perceived as a matter of concern by all cancer patients (Borràs *et al*, 2010), as is indeed recognised in the Catalonian Cancer Plan (2009).

## Figures and Tables

**Table 1:** Detailed breakdown of the 83 professionals interviewed

From an institutional standpoint	Professionals who received the order to implement the fast track	Medical director	9	16
		Programme coordinator (clinician)	7	
From a technical standpoint	Professionals who have led organisational change towards fast-track development	Clinical leader (by tumour site)	19	22
		Epidemiologist or quality analyst	3	
From a process standpoint	Professionals usually working with this type of organisational approach	General practitioner	11	38
		Specialist	12	
		Nurse case manager	12	
		Secretary or documentalist	3	
Planning professionals from the health regions				7

**Table 2:** Monitoring indicators by type of cancer (2006–2009)

**Tumour site**	**Year**	**Patients included in the CFP (*n*)**	**Cancer diagnosed through CFP route** a	**Patients referred from GP** a	**Compliance with referral guidelines** a	**Cancer detection rate** a	**Time between suspect detection–treatment start (mean days)**
Colorectal cancer	2006	3642	42.0 (40.4–43.7)	60.1 (58.2–61.4)	77.3 (74.8–77.6)	40.7 (39.2–42.3)	30.4
	2007	5903	50.1 (48.5–51.7)	61.1 (59.9–62.4)	76.1 (75.0–77.2)	32.9 (31.7–34.1)	29.1
	2008	6786	45.2 (43.8–46.6)	59.6 (58.4–60.7)	77.0 (76.0–78.0)	29.3 (28.3–30.4)	27.1
	2009	8077	54.3 (52.9–55.7)	60.7 (59.6–61.9)	80.6 (79.8–81.4)	28.7 (27.7–29.7)	29.6
							
Lung cancer	2006	3363	60.2 (59.8–63.4)	60.6 (59.0–62.3)	70.8 (69.1–72.1)	49.9 (48.2–51.6)	30.8
	2007	2819	51.8 (50.0–53.7)	47.1 (45.3–49.0)	71.2 (69.5–72.8)	52.9 (51.1–54.7)	38.9
	2008	3662	46.6 (45.0–48.2)	49.7 (48.1–51.3)	85.5 (84.4–86.7)	44.0 (42.5–45.6)	32.25
	2009	3841	53.2 (51.5–54.9)	41.4 (39.7–42.9)	82.3 (81.1–83.5)	39.7 (38.1–41.2)	36.7
							
Breast cancer	2006	1581	38.4 (38.5–42.5)	48.3 (46.6–51.5)	81.1 (79.4–83.2)	51.5 (49.1–54.0)	35.7
	2007	5225	60.4 (58.9–62.0)	52.4 (51.0–53.7)	86.5 (85.6–87.5)	45.0 (43.7–46.3)	31.8
	2008	5416	56.8 (55.3–58.3)	54.1 (52.8–55.4)	92.3 (91.6–93.0)	40.2 (38.9–41.5)	31.5
	2009	5705	58.2 (56.7–59.6)	42.8 (41.5–44.1)	87.9 (87.0–88.7)	44.0 (42.7–45.2)	32.1

Abbreviations: CFP=Cancer Fast-track Programme; GP=general practitioner.

a Proportion (CI95%).

**Table 3:** Organisational innovation along the different stages of the fast-track process

**Specific changes**	**Key objectives**
*From suspected cancer detection to confirmation of diagnosis*	
Clinical discussion of guidelines in multidisciplinary groups of both levels of care	
Generation and dissemination of information	High degree of compliance with clinical guidelines
Review and updating of inclusion criteria	
Unification of hospital-access gateways	Effective referral to diagnosis between care levels
Direct electronic access to outpatient appointment or a single clear pro-forma	
Discussion of referral track by clinicians and data-processing staff of both levels (to prevent lags as a result of administrative errors)	
	
*From confirmation of diagnosis to first treatment*	
Protocolisation of diagnostic tests	
Establishment of a ‘triple priority’, that is, rapid diagnosis of high, low probability and ordinary list	Improving the queuing mechanisms for accessing services
Slots in schedules for diagnostic tests and rechanelling to the ordinary list in the event of cancellation	
Operating-theatre slottings	Preventing operating theatre bottle–necks
Extension of knowledge of referral guidelines and referral track to all possible origins of suspicions at the hospital	Effective referral to diagnosis between clinical departments
Case management (notification of referrals, patient counselling, coordination of appointment schedule and tumour committee role)	Improving coordination and speed of processes
